# Effects of initial foot position on neuromuscular and biomechanical control during the stand-to-sit movement: Implications for rehabilitation strategies

**DOI:** 10.1371/journal.pone.0315738

**Published:** 2025-02-14

**Authors:** Woohyoung Jeon, Ashley Dalby, Xuanliang Neil Dong, Chung-Hyun Goh

**Affiliations:** 1 Department of Health & Kinesiology, University of Texas at Tyler, Tyler, TX, United States of America; 2 Department of Mechanical Engineering, University of Texas at Tyler, Tyler, TX, United States of America; New Jersey Institute of Technology Newark College of Engineering, UNITED STATES OF AMERICA

## Abstract

**Background:**

Initial foot position (IFP) is one of the important movement strategies that influence neuromuscular and biomechanical control during sit-to-stand (STS) movements. Similarly, stand-to-sit (StandTS) is vital in rehabilitation settings for evaluating strength and balance control during descending movements. Understanding how IFP impacts changes in biomechanical and neuromuscular movement control factors during StandTS can provide valuable insights for designing effective rehabilitation programs.

**Methods:**

Twelve healthy young adults participated in this study, examining three symmetric IFPs: (1) REF (reference); (2) wide: each foot was shifted outwards by 30% from REF; (3) TO (toes-out): symmetric toes-out angle of 30° from REF. Kinematic and kinetic differences among the three IFPs during StandTS were analyzed, along with the characteristics of muscle activation patterns using muscle synergy analysis.

**Results:**

In the wide IFP, trunk flexion angle was reduced, and valgus angle was greater than in the other IFPs. The TO IFP resulted in greater dorsiflexion and knee flexion angles compared to the REF and wide IFPs. Compared to the REF IFP, both wide and TO IFPs showed greater eccentric work at the hip joint in the anterior-posterior (power absorption) and the vertical direction (gravitational force control) and demonstrated reduced postural sway in medio-lateral and vertical directions. Muscle synergy analysis of EMG activity revealed increased activation of back and plantar flexor muscle in the wide IFP, and increased contribution of hip joint muscles in the TO IFP.

**Conclusion:**

The wide IFP increased the valgus angle, leading to reduced trunk flexion with increased back muscle activation. The TO IFP enabled greater angular displacement at the ankle and knee joints, enhancing hip joint muscle involvement in StandTS movement control. Both the wide and TO IFPs facilitated hip joint work, improving postural sway control during the descent phase of StandTS. These findings provide valuable insights for designing rehabilitation strategies tailored to specific patient needs.

## Introduction

The sit-to-stand (STS) movement is a fundamental daily activity requiring coordinated neuromuscular and biomechanical control mechanisms [1,2]. Therefore, STS motion is widely used in rehabilitation settings to assess the strength, postural stability, and mobility of older adults [1,3]. Initial foot position (IFP) is a critical factor in optimizing these control mechanisms, as it determines force distribution across the lower limbs and affects joint angles, muscle activation patterns, and overall movement dynamics [4–6]. Extensive research on STS highlights the importance of IFP as an initial movement strategy to enhance stability and reduce fall risk, particularly in populations with impaired mobility, such as frail elderly or patients with neurological disorders [4,6–8].

While the STS has garnered considerable attention, the reciprocal stand-to-sit (StandTS) movement is equally vital but less studied. StandTS involves a controlled descent from standing to sitting, which is also integral to daily life and is used in rehabilitation to assess descending balance control and lower limb strength [9,10]. Although StandTS may initially appear to be a reverse movement of STS, the necessary force control and associated muscle activation patterns differ fundamentally from those in STS. StandTS necessitates precise muscle coordination and joint movement to maintain balance [10].

In contrast to the predominant role of knee extensor concentric contraction in the rising phase of STS to generate vertical force [4,5], StandTS demands a distinctive aspect of motor control for descending balance control, particularly from the hip and knee joints. To maintain the center of mass (CoM) within the base of support (BoS)–the area between the feet–StandTS requires precise, coordinated neuromuscular activity across several joints and muscle groups to control the speed of descent and ensure a smooth, safe landing [10]. This specific motor control strategy is essential for avoiding falls and ensuring stability during the downward movement.

During the StandTS, eccentric muscle activation by the knee extensors, hip extensors, and ankle plantarflexors control the descending movement [9–11] These muscles contract eccentrically as the hips move backward and down, the knees bend while the ankles adjust to maintain balance to slow the body’s descent and control the downward motion to prevent a sudden fall into the seated position [11–13]. Therefore, proper joint coordination through eccentric control at the ankle, knee, and hip joints is essential to maintain balance. Additionally, postural adjustments of upper body through the core and trunk muscles are required to ensure the spine remains in a neutral and stable position during the transition as CoM shifts backward to avoid postural instability [14].

Using a raised chair or surface is an effective initial movement strategy to improve postural stability during the STS and StandTS [15], as it reduces the load on lower body muscles, allowing for better focus on balance control. Changes in IFP during both STS and StandTS can also serve as an initial movement strategy, as IFP significantly influences biomechanical and neuromuscular factors, including force dynamics at the joints and the supporting muscle activation pattern [8–10,16].

Understanding the role of IFP during StandTS is crucial for several reasons. Variations in IFP can lead to different kinematic and muscle activation patterns when sitting down, resulting in varied kinetic outcomes. This flexibility allows individuals to effectively control their descent according to their goal by adjusting IFP during StandTS. For example, a wide or toes-out (TO) IFP provides a broader BoS, affecting joint angles and muscle recruitment patterns differently compared to a neutral (standard) position [4]. These differences impact balance, stability, and muscle workload during the movement. With a TO IFP, the alignment of the knee relative to the hip and foot changes, creating a more open angle at the hip joint [4,6]. This shift can distribute more load onto the lateral aspects of the hip and thigh muscles, prompting changes in muscle activation patterns.

Therefore, in clinical and rehabilitation contexts, optimizing IFP can enhance therapeutic exercise efficacy, improving balance and functional strength. Personalized IFP adjustments can cater to individual biomechanical needs. For example, optimized the IFP during StandTS to engage more hip muscles can strengthen the hip joint and improve descending balance. This detailed approach can lead to better outcomes in rehabilitation programs tailored to diverse populations, including older adults and individuals recovering from lower limb injuries.

Despite this recognized importance of IFP in StandTS, there is a lack of research specifically addressing its impact on StandTS movements. Therefore, this study aims to fill this gap by analyzing how different IFPs affect kinematics, kinetics, and muscle activation patterns during StandTS. We hypothesize that varying IFPs will lead to distinct differences in joint angles, muscle activation patterns, and balance control during StandTS. Specifically, we anticipate that a wide or TO IFP will enhance hip joint engagement and modify the dynamics of ankle and knee movements, thereby contributing to improved postural stability during the StandTS descent.

## Materials and method

### Participants

Twelve healthy younger adults participated in this study (Table 1). Physical activity level (the number of days and hours spent walking and doing physical activities per week) was measured using the International Physical Activity Questionnaire [17].

**Table 1:** Anthropometrics characteristics of study participants.

Characteristics	Male (n = 6)	Female (n = 6)	*P*—value
**Anthropometric**			
Age (years)	21.33 ± 1.86	21.66 ± 1.63	0.74
Height (cm)	174.20 ± 7.89	165.48 ± 6.53	0.06
Weight (kg)	74.68 ± 6.97	65.70 ± 5.74	0.04*
BMI (kg/m^2^)	24.56 ± 0.32	23.97 ± 1.19	0.26

Values are presented as mean ± standard deviation.

* represents a significant difference between male and female, determined by an independent samples t-test.

Participants were included in this study if they had a “moderate” or higher physical activity level, which meets the following criteria: a) three or more days of vigorous-intensity activity of at least 20 minutes per day OR b) five or more days of moderate-intensity activity and/or walking of at least 30 minutes per day OR c) five or more days of any combination of walking, moderate-intensity or vigorous intensity activities achieving a minimum total physical activity of at least 600 minutes/week.

Participants were excluded from this study if they had 1) deficits or disorders that could affect balance control; 2) history of dizziness and imbalance; 3) history of neurological (e.g., Parkinson’s disease, Alzheimer’s disease, stroke, visual and/or vestibular impairment), musculoskeletal, or any other systemic disorders; and 4) Body Mass Index (BMI) within the overweight and obesity range (BMI is higher than 25 kg/m^2^).

The principal investigator (PI) and his research assistants explained the purpose, research procedures, potential risks, and benefits to the prospective participants. The potential participant was asked to read the informed consent form before being allowed to participate, ensuring that they understood the presented information. PI and his research team members were on hand to oversee each participant during the test and conducted data collection according to the data collection protocol. All procedures were approved by the Institutional Review Board at the University of Texas at Tyler (IRB#: 2022–118) and dated October 4, 2022, and were in accordance with the Helsinki Declaration of 1975. All participants provided written informed consent prior to study participation. The start and end of the recruitment period for this study is from 18/08/2023 to 28/05/2024.

### Data collection

**Fig 1:**
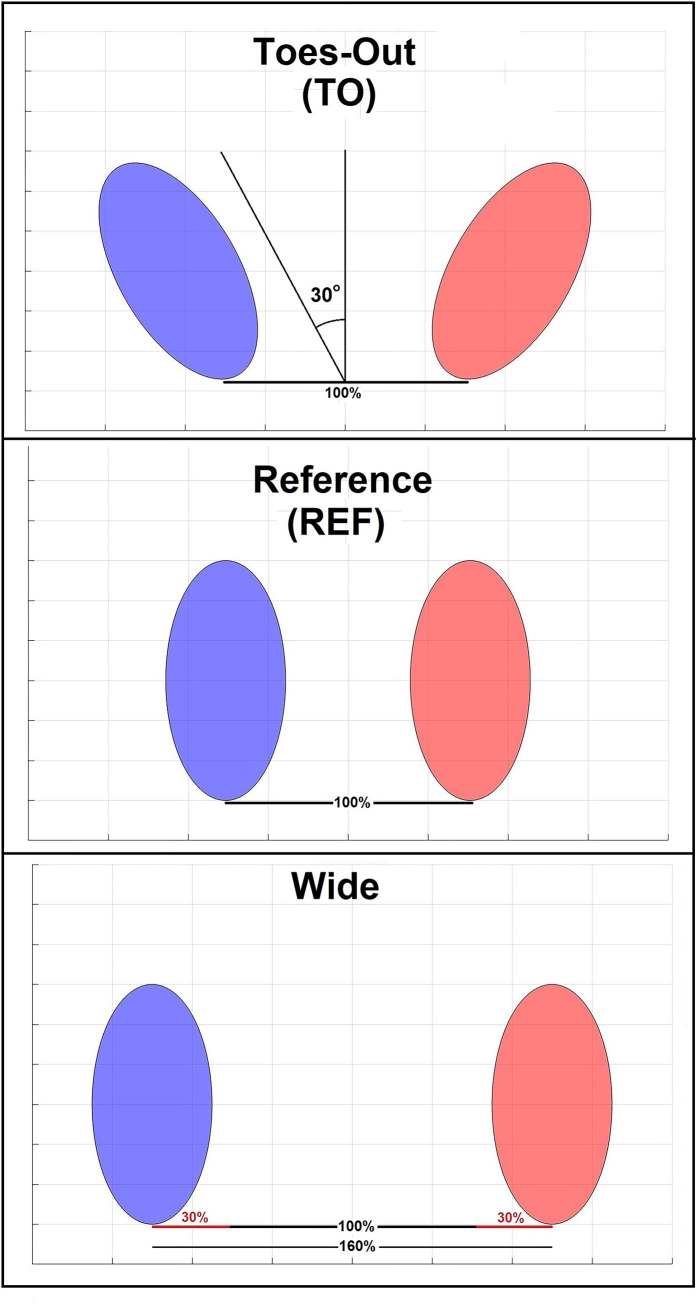
The three different initial foot positions (IFPs) tested. (1) REF: Reference; (2) wide: Each foot was shifted outwards 30% from REF; (3) TO (toes-out): Symmetric toes-out angle of 30° from REF.

For StandTS testing, participants maintained an upright standing position with their feet shoulder-width apart and both feet on a force plate (Bertec Corp., Columbus, OH). The three symmetric initial foot positions (IFPs) tested were: (1) reference (REF): participants stood in a self-selected shoulder width stance (defined as 100%) with feet in a parallel position. (2) wide: participants were standing with a wide stance, each foot was shifted laterally 30% of the shoulder width from REF, and (3) toes-out (TO): Participants turned their toes laterally 30° from REF (Fig 1).

During the StandTS task, participants stood with their arms crossed over their chest in the required IFP. Once the light cue turned on, they sat down on an armless, backless bench, ensuring the backs of their knees did not touch the bench. The seat height was individually adjusted for each participant based on the distance from the center of the knee joint to the floor, achieving a 90-degree knee angle when seated.

Upon activating a visual light cue, participants performed the StandTS task from three different IFPs (REF, wide, TO) at their preferred speed. A pressure-sensitive force sensor pad (Smart Caregiver, USA) on the seat was used to determine the initiation point of sitting based on the magnitude and distribution of sitting pressure.

In the practice session, participants practiced StandTS once in each IFP (REF, wide, and TO). In the test session, five trials were performed for each of StandTS condition in random order. Following completion of each StandTS trial, participants maintained the seated position for 5 seconds.

The test lasted approximately for 2 hours on average and was completed in a single day. All 12 subjects completed the test without any attrition.

#### Kinetics and kinematics

The force plate recorded center of pressure (CoP) and ground reaction force (GRF) on the anterior-posterior (A-P), medio-lateral (M-L), and vertical axes. GRF was normalized to body mass (kg). The force plate data was collected at 1000 Hz.

The full body kinematics was recorded using the Vicon motion analysis system (Oxford Metrics Group Ltd., Oxford, UK). Thirty-nine reflective markers were placed on anatomical landmarks based on the full-body modeling (Vicon Nexus 2.12) with Plug-in-Gait (Vicon Motion System, Oxford Metrics Group Ltd). Motion capture data was collected at 100 Hz.

#### EMG

A wireless EMG System (BIOPAC Co., USA) was used for the acquisition of muscle activity signals. BIOPAC adhesive pre-gelled Ag/AgCl surface EMG electrodes (size: 11 mm diameter, 35 mm vinyl backing) were placed bilaterally on the tibialis anterior (TA), medial gastrocnemius (mGas), vastus lateralis (VL), biceps femoris (BF), gluteus maximus (Gmax), gluteus medius (Gmed), and erector spinae (ES). The positioning of the electrodes was in accordance with SENIAM guidelines [18]. The VL is selected among the quadricep muscles (vastus medialis (VM), vastus intermedius (VI), and rectus femoris (RF)) because it is the strongest member of the group and a key contributor to anterior knee pain syndromes [19]. The VL is estimated to produce about 40% of the overall strength of the quadriceps, while the RF and VI contribute to 35% and the VM account for the remaining 25% [20]. To normalize EMG amplitude for each muscle, participants performed maximum voluntary isometric contraction (MVIC) for each muscle before collecting data [21].

For the TA and mGas, maximal dorsiflexion and plantarflexion were performed, respectively, with the foot strap (ankle and knee joints at 90°). For the VL, maximal knee extension was performed with the participant seated on a chair (hip and knee joints at 90°), resisting external manual force applied around the ankle. For the BF, participants lay on a table and performed maximal knee flexion at 45° knee flexion against manual resistance at the ankle. For Gmax and Gmed, participants lay prone and side-lying, respectively (hip and knee joints at 0°), performing maximal hip extension and abduction against manual resistance at the ankle. For the ES, participants lay face-down on a flat mat with their palms covering their eyes. While their legs were held by an assistant, they were instructed to lift their upper body as high as possible [5]. They performed maximal hip extension against manual resistance at the ankle joints. EMG data were sampled at 1000 Hz.

### Data processing

**Fig 2:**
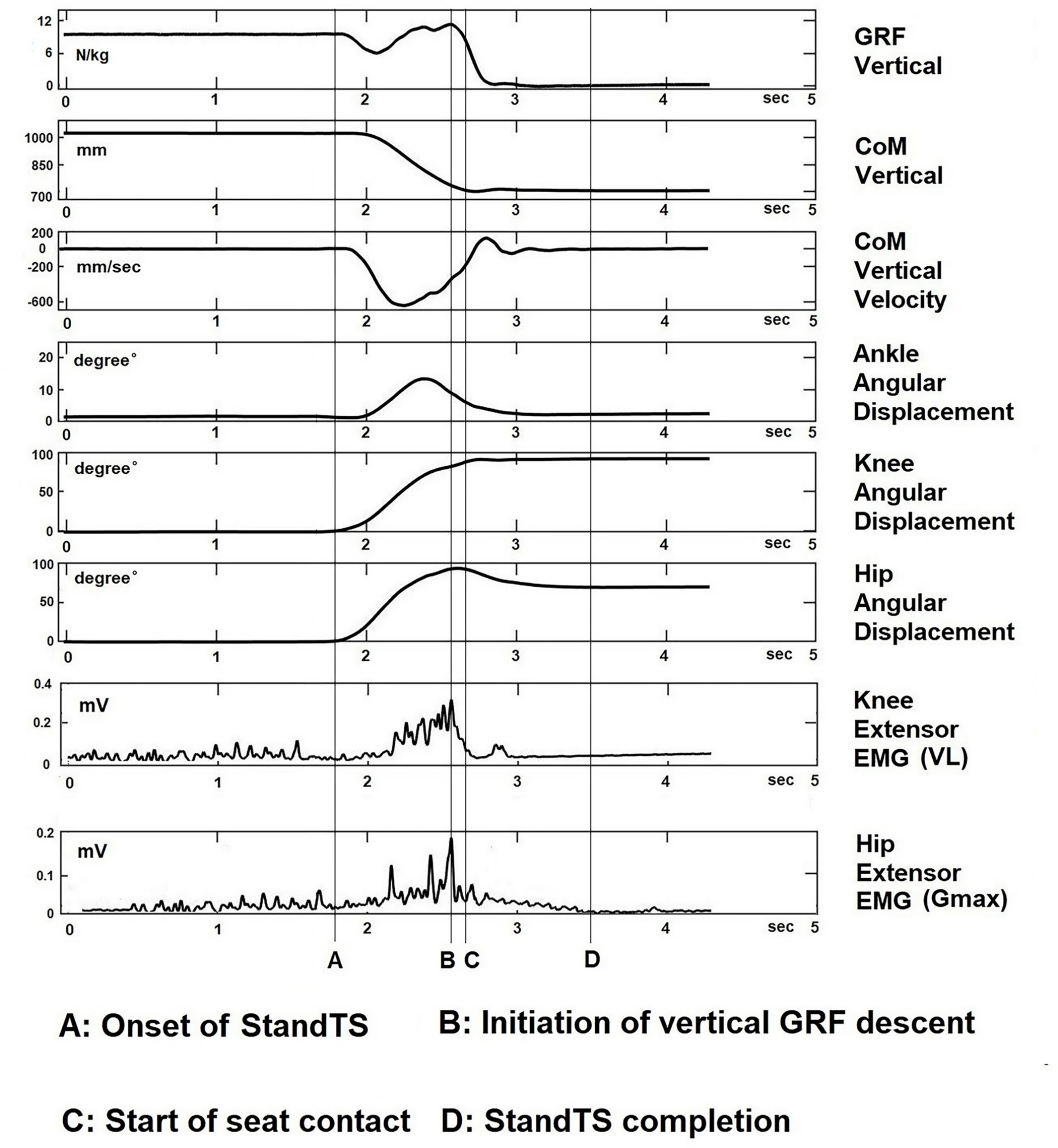
Kinematics of Stand-to-sit in reference initial foot position from a representative subject. The distinctive feature of the knee extensor eccentric control mechanism was observed in its continuous activation increase from the onset of StandTS until right before the CoM vertical dropping (vertical GRF descent) (from time point A to time point B, controlled descending phase).

All kinetics, kinematics, and EMG variables during the descending phase of StandTS were computed using a custom-written algorithm in MATLAB (version 2023b, The Mathworks Inc, Natick, MA, USA). The descending phase of StandTS was defined as spanning from the onset of StandTS to just before the initiation of vertical GRF descent. This phase highlights that the knee extensor remains activated until the last minute of the “controlled” descending phase. After this timing point, leg muscle activation is released for the accelerated descent into sitting (Fig 2, time point B). Beyond this point (Fig 2, between time points B and C), the descent is characterized by a rapid drop of center of mass (CoM) until seated, no longer controlled by knee extensor eccentric control mechanism (Fig 2, seventh panel). The average of five trials for each IFP was calculated for data analysis.

#### Kinetics and kinematics

The body’s center of mass (CoM) trajectory, joint angular displacement and power were calculated using Vicon Nexus 2.12 Software (Vicon, Oxford Metrics, UK) and a custom-written algorithm in MATLAB (version 2023b, The Mathworks Inc, Natick, MA, USA). Trunk flexion angle was defined as the angle between the thorax and the laboratory coordinate system specified in the Plug-in Gait Model from Vicon Nexus. Kinematic and kinetic data were low-pass filtered through a fourth-order Butterworth filter with a cut-off frequency of 6 Hz and 25 Hz, respectively [4].

Valgus angle: The valgus angle before sitting down was calculated by following steps:

When the given coordinates are:

Normal = Hip: [0,0], Knee: [0, −10], Ankle: [0, −20],

Valgus = Hip: [0,0], Knee [0, −10], Ankle [−3, −20])

1. Using the coordinates:
  $\vec{v}f$ = knee − hip = [0,−10] − [0,0] = [0,−10], $\vec{v}t$ = ankle–knee = [−3,−20] − [0,−10] = [−3,−10]
2. Calculate the angle of each vector with respect to the horizontal axis:
  The angle θf of the femur vector $\vec{v}f$: θf = atan2 ($\vec{v}f$ [2], $\vec{v}f$ [1]) = atan2 (−10, 0)
  The angle θt of the tibia vector $\vec{v}t$: θt = atan2 ($\vec{v}t$ [2], $\vec{v}t$ [1]) = atan2 (−10, −3)
3. Calculate θf: θf = atan2(−10, 0) which corresponds to −90° (or 270° for positive direction).
4. Calculate θt: θt = atan2(−10, −3)
  θt = atan2(−10, −3) ≈ −106.7°
5. Find the difference between the angles to get the valgus angle:
  Valgus angle α = θt–θf = −106.7°−(−90°) = −106.7° + 90° = −16.7°

**Fig 3:**
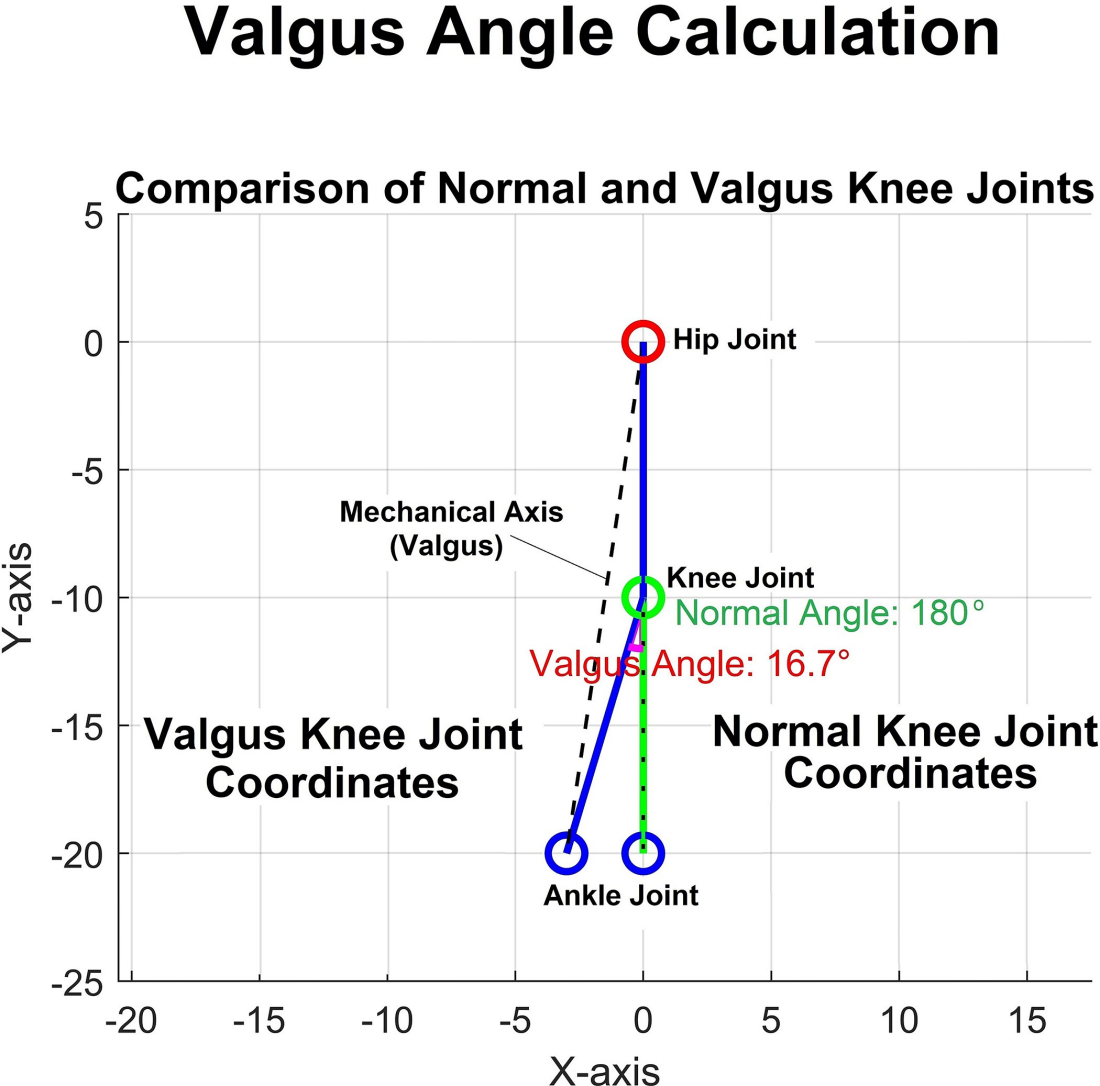
Valgus angle calculation.

Since the angle we are interested in should be positive, consider the absolute value:

α = ∣−16.7°∣ = 16.7°. Therefore, the calculated valgus angle is approximately 16.7° (Fig 3).

Joint Work: Work at the knee and hip joints during StandTS: Negative eccentric work in the anterior-posterior (AP) direction—representing ground impact power absorption—and positive eccentric work in the vertical direction—representing resisting gravity during StandTS—were calculated by the integration of the power-time curve (see Fig 6, third and bottom panels). All work data were normalized to body weight (J/kg).

Postural sway: Measures of the mean velocity of CoP were used to quantify postural sway in the A-P and M-L directions during StandTS [4,5,22]. Measure of the standard deviation of the body’s center of mass acceleration (SDCoMAccel) was used to quantify postural sway in the vertical direction during StandTS [4,23].

#### EMG

The raw surface EMG (sEMG) data collected during StandTS underwent the following processing steps:

1. Pre-processing of sEMG signal: Any DC offset was first eliminated using the “detrend” function in MATLAB. Next, a median filter was applied to the signal to remove noise [24], followed by the application of a 20- 450Hz bandpass filter to extract the frequency range where muscular energy is concentrated [25].
2. sEMG rectification and linear envelope: sEMG signal values below zero were converted to positive values of the same amplitude to create a full-wave rectified sEMG signal (see Fig 6, top panel). To obtain sEMG envelopes, a 2nd order Butterworth low pass filter with a 20 Hz cutoff frequency was applied for digital smoothing. This filtered out high-frequency noise, enhancing the signal-to-noise ratio and improving clarity for identifying underlying patterns [21].

Muscle Synergy: To analyze muscle patterns for balance recovery, we used non-negative matrix factorization (NNMF) on the sEMG data from StandTS trials. We created sEMG envelopes using a moving root mean square (RMS) with a window length of 100 samples to better capture the intensity of muscle activation and identify patterns in muscle recruitment [26]. The synergies were extracted from five trials of each StandTS IFP from the dominant leg muscles during the descending phase.

Extraction of muscle synergy: NNMF decomposed the EMG signals into two components: muscle-weighting and temporal synergy activation from the EMG matrix [27,28], representing the muscle patterns during the descending phase of StandTS:

1. W: This vector specifies the spatial pattern of the relative activation level of each muscle in the muscle synergy. Each muscle’s contribution is relatively weighted within this spatial structure.
2. C: This scaling coefficient represents the temporal synergy activation, specifying how the coordinated muscle activation pattern is modulated over time during the targeted movement period.

The spatial components are multiplied by a scaling (synergy recruitment) coefficient C. This transformation can be expressed as:

$$EMG_{0}(m\times t)=W(m\times n)\cdot C(n\times t)+e=EMGr(m\times t)+e,$$ (1)

(where m = the number of muscles, t = the number of time points, n = the number of muscle synergies, e = residual error, and EMG_0_ = observed EMG signal matrix, and EMGr = reconstructed EMG signal matrix)

To evaluate the similarity between EMG_0_ and EMGr, variability accounted for (VAF) was calculated according to the following equation:

$$VAF=\left(1-\frac{{\left({EMG}_{0}–{EMG}_{r}\right)}^{2}}{{\left({EMG}_{0}-mean({EMG}_{0})\right)}^{2}}\right)\times 100\%$$ (2)

To determine the optimal number of synergies, we repeated the optimization algorithm, updating the synergy weights and activation coefficients in each iteration. For each iteration, we calculated the VAF to assess the quality of the reconstructed EMG signals. The optimal number of synergies (k) was chosen as the smallest value that yielded a VAF greater than 90% [29].

### Statistical analysis

A statistical software (IBM SPSS Statistics 25; Chicago, IL, USA) and a custom-written algorithm in MATLAB were used for all statistical analyses, with an established a-priori alpha level of 0.05. For the justification of our sample size, an a priori power analysis was conducted using G*Power [30]. Effect size (Cohen’s d) was calculated based on previous studies [9,10,13,21,31]. We detected an effect size of 0.78. Through power calculation, we determined that with 12 participants, there would be 80% power (1 –β) at a 5% level of significance (α). The Shapiro-Wilk test was used to assess the normality of the kinematics, kinetics, and EMG data.

The mean of five trials of each StandTS condition (REF, wide, TO) was used for all kinematics, kinetics, and EMG analyses. To confirm if the variance between the five trials within each IFP was significantly smaller than the variance between the three IFPs, we conducted a one-way ANOVA. This analysis tested whether the differences between the three StandTS conditions (REF, wide, TO) were significantly greater than the variance observed within each StandTS condition across the five trials.

A one-way repeated measures ANOVA was used to determine differences in 1) the angular displacement of the ankle, knee, hip, and trunk flexion; 2) valgus angle at beginning of StandTS 3) knee and hip extensor eccentric work in the AP and vertical directions; and 4) postural sway (mean velocity of CoP) in the AP and medio-lateral (ML) directions, along with standard deviation of center of mass acceleration (SDCoMAccel) in the vertical direction among the three IFPs (REF, wide, TO).

The k-mean clustering algorithm (from MATLAB R2023b) categorized the similar groups of muscle synergies extracted from EMG activity during StandTS across all participants. Subsequently, intra-class correlation coefficient (ICC) was used to examine the internal consistency of all muscle synergies. Common muscle synergies shared among three IFPs (REF, wide, TO), with an ICC over 0.75 (indicating good reliability), were categorized in the same cluster. For identifying synergies specific to either the REF, wide, and TO conditions, muscle synergies with an ICC value over 0.9 (indicating excellent reliability), were categorized in the same cluster [32].

## Results

All data are presented as mean ± standard deviation (SD). All kinematics, kinetics, and EMG data were normally distributed. The descending phase of StandTS were analyzed. The average of the variance of the five trials within each IFP was significantly smaller than the average of the variance between the three IFPs (*p* < 0.01).

### Angular displacement of the ankle, knee, hip, and trunk

**Fig 4:**
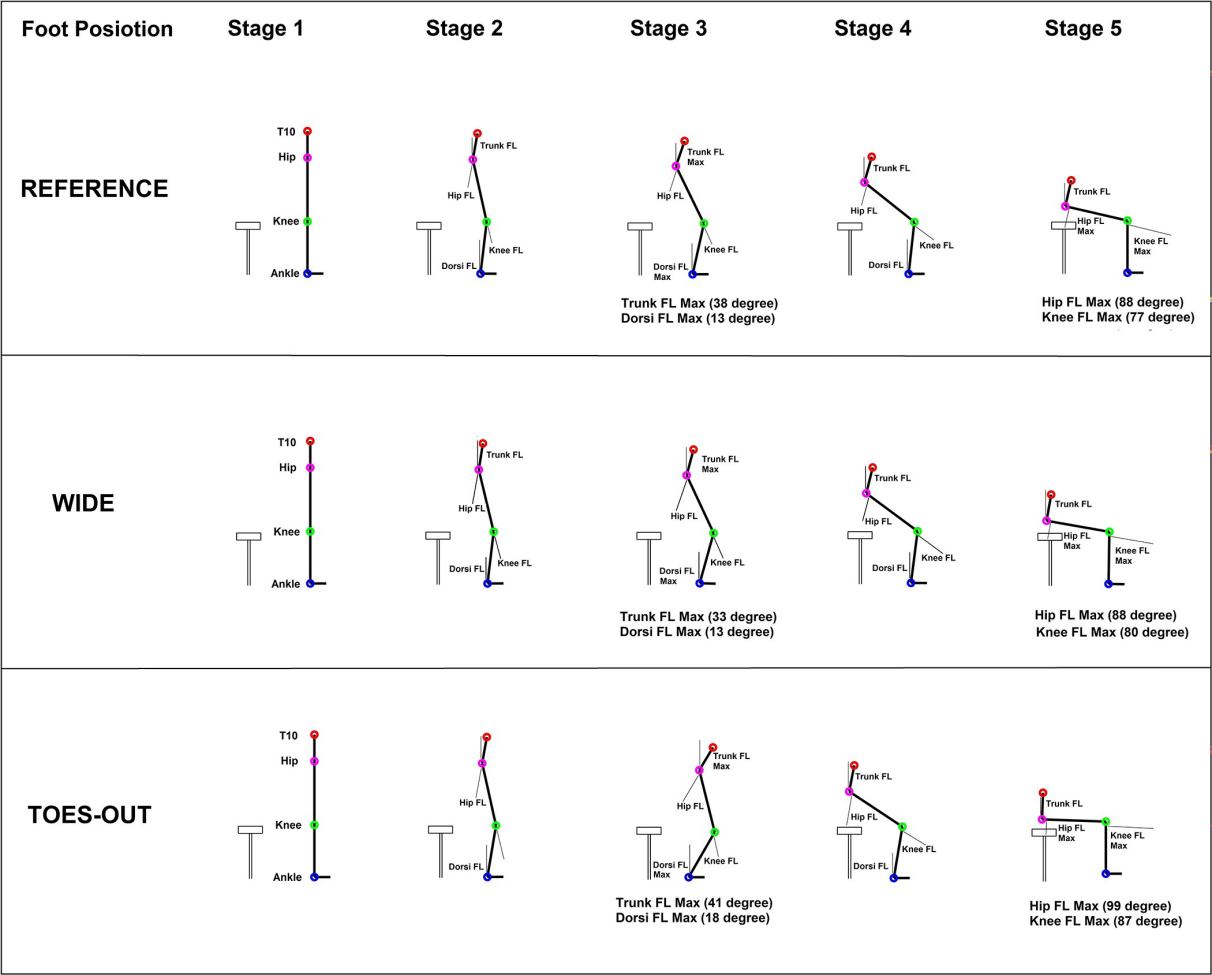
Angular displacement of the ankle, knee, hip, and trunk during stand-to-sit (StandTS). Stick figures illustrate the degree of flexion at each stage of StandTS (Stage 1: Initiation of StandTS, Stage 5: The last moment of ‘controlled’ descending motion right before rapidly dropping to the seat, Stage 3: The midpoint between Stage 1 and Stage 5, Stage 2: The midpoint between Stage 1 and Stage 3, and Stage 4: The midpoint between Stage 3 and Stage 5). TO IFP showed greater dorsiflexion and knee flexion than REF and wide, while wide showed smaller trunk flexion than REF and TO.

There was a main effect of the IFP on ankle dorsiflexion and knee flexion during StandTS. In TO, greater dorsi flexion (18.09° ± 5.22°, *p* < 0.01, effect size: η2 = 0.61, observed power = 0.90) and knee flexion (87.74° ± 9.80°, *p* < 0.01, effect size: η2 = 0.66, observed power = 0.95) were observed (Fig 4, stage 3 and Fig 7, top panel (A)) compared to REF (Dorsiflexion: 12.53° ± 4.72°, Knee flexion: 76.21° ± 7.70°) and wide (Dorsiflexion:13.64° ± 3.07°, Knee flexion: 80.12° ± 8.64°) (Table 2).

**Table 2:** Angular displacement of the ankle, knee, hip, and trunk and valgus angle during stand-to-sit (StandTS).

IFP	Ankle Dorsiflexion (°)	Knee Flexion (°)	Hip Flexion (°)	Trunk Flexion (°)	Valgus Angle (°)
**REF**	12.53 ± 4.72	76.21 ± 7.70	89.87 ± 13.07	38.68 ± 8.11	5.17 ± 2.24
**Wide**	13.64 ± 3.07	80.12 ± 8.64	100.50 ± 12.63	33.17 ± 5.55*	8.48 ± 3.07*
**TO**	18.09 ± 5.22*	87.74 ± 9.80*	99.14 ± 10.92	40.62 ± 7.21	4.21 ± 1.95

Values are presented as mean ± standard deviation.

* represents a significant difference from the other two IFPs.

No significant differences were found in hip flexion among the three IFPs (Fig 4, stage 5 and Fig 7, top panel (A)). Additionally, the wide IFP showed significantly smaller trunk flexion (33.17° ± 5.55°, *p* = 0.02, effect size: η2 = 0.52, observed power = 0.78) compared to REF (38.68° ± 8.11°) and TO (40.62° ± 7.21°) (Fig 4, stage 3 and Fig 7, top panel (A)).

### Valgus angle

**Fig 5:**
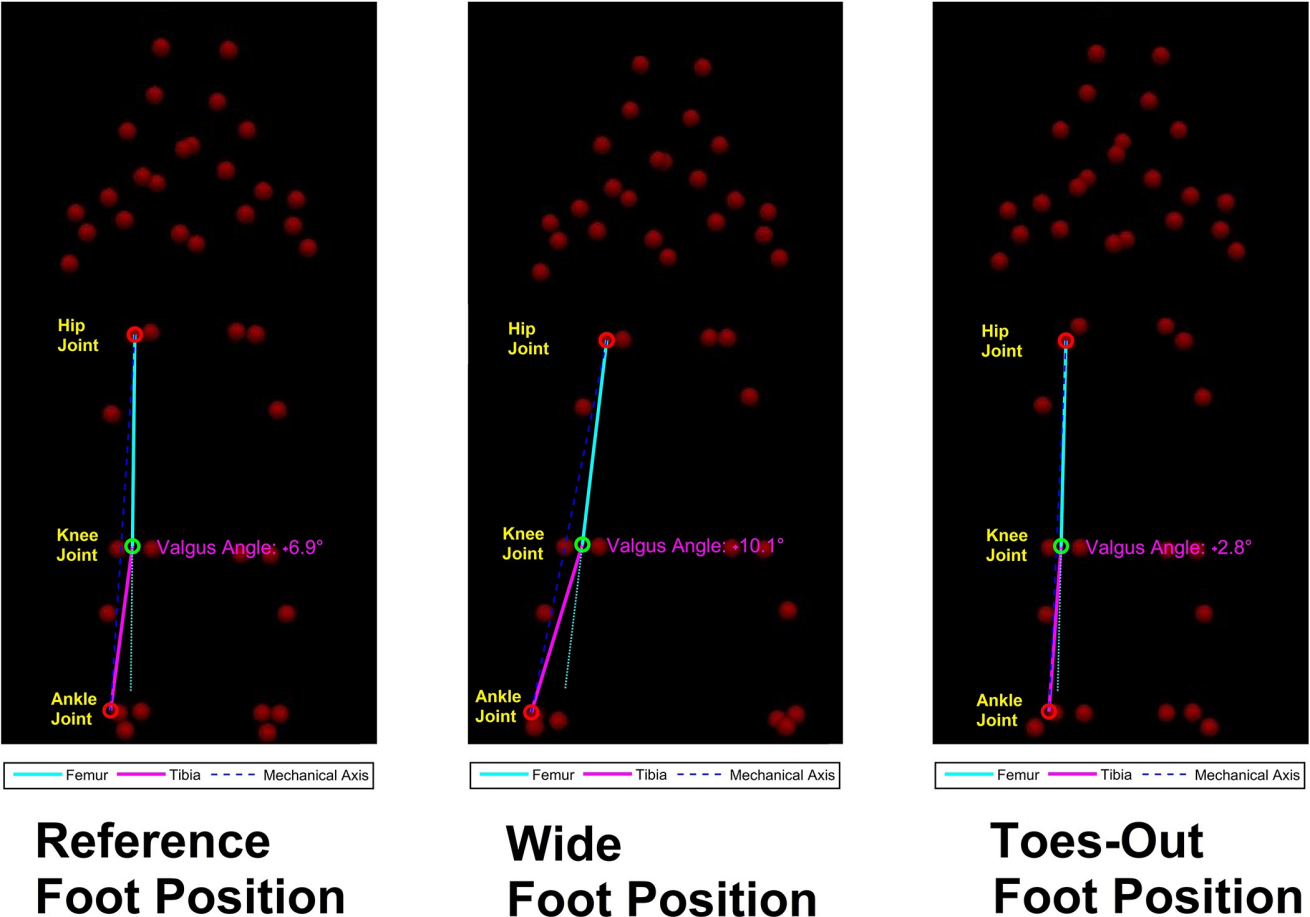
Valgus angle differences among REF, wide, and TO. The wide IFP showed significantly greater valgus angle at the beginning of StandTS.

There was a main effect of the IFP on the valgus angle at the StandTS initiation. In wide, a greater valgus angle (8.48° ± 3.07°, *p* < 0.01, effect size: η2 = 0.64, observed power = 0.90) was observed (Fig 5) compared to REF (5.17° ± 2.24°) and TO (4.21° ± 1.95°) (Figs 5 and 7, top panel (A)) (Table 2).

### Knee and hip joint work

**Fig 6:**
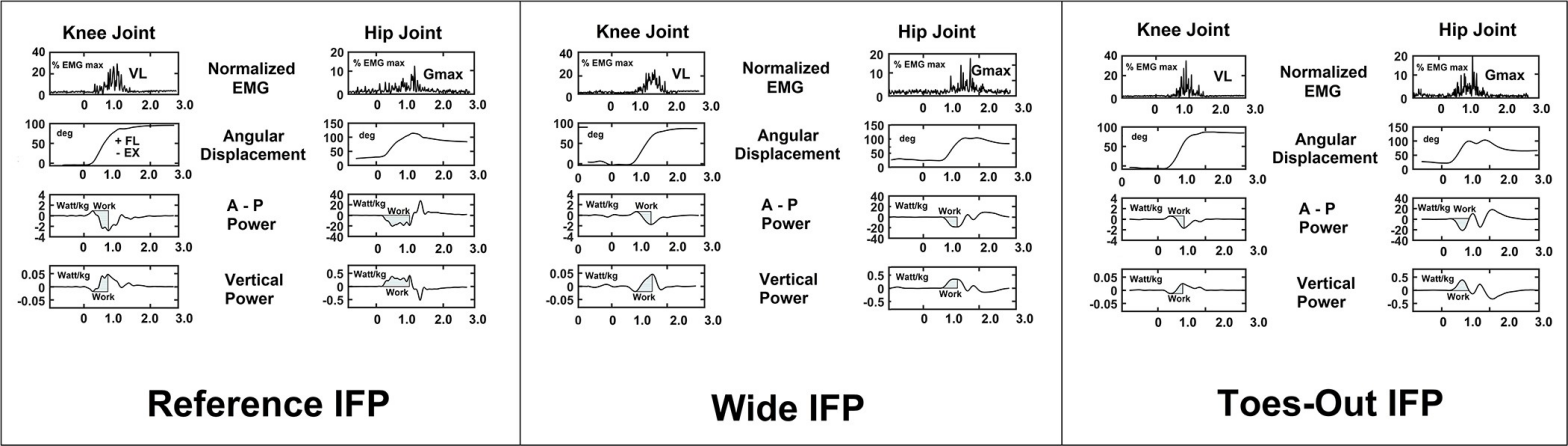
Work calculation in each IFP from a representative participant. At the knee and hip joints, work during the descending phase was calculated by integrating the power-time curve. The third panel illustrates negative eccentric work in the anterior-posterior (AP) direction for ground impact absorption, while the fourth panel depicts positive work in the vertical direction for resisting gravity and controlling descent until just before seating.

Work at the knee and hip joints (eccentric joint work) during StandTS was separated into two based on the direction of movement. Negative eccentric work in the AP direction is responsible for ground impact power absorption, while positive eccentric work in vertical direction is involved in resisting gravity to control the descending speed during StandTS (Fig 6).

**Fig 7:**
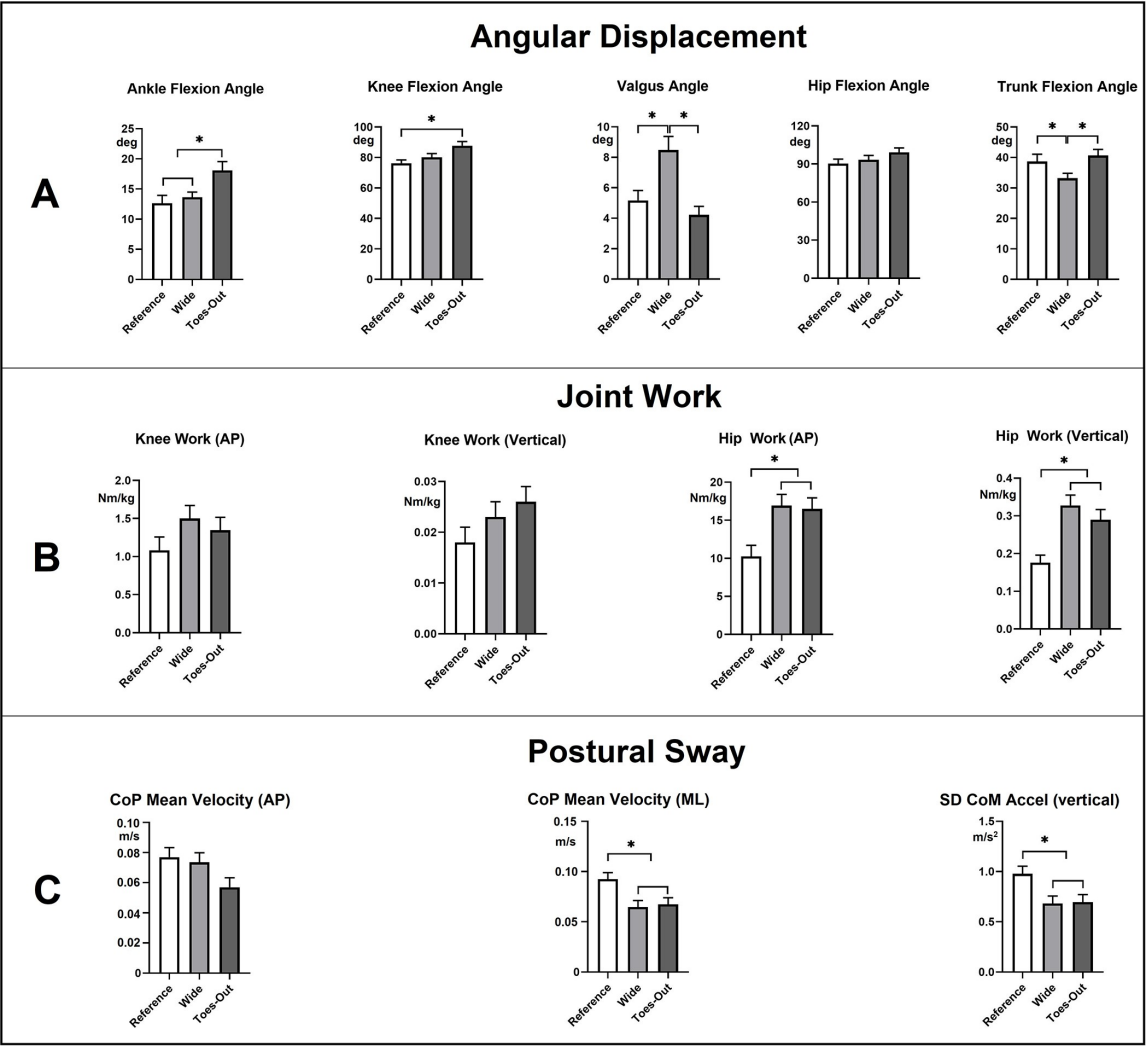
Differences in angular displacement (A), eccentric joint work (B), and postural sway (C) among REF, wide, TO IFPs during the descending phase of stand-to-sit. Deg: Degree, CoP: Center of pressure, AP: Anterior-posterior, ML: Medio-lateral, SDCoMAccel (standard deviation of center of mass acceleration). * indicates significantly difference, (*p* < 0.05).

At the knee joint, there was no significant difference in eccentric work among the IFPs in both the AP and vertical directions. However, at the hip joint, the wide and TO IFPs demonstrated significantly greater eccentric work than the REF in both AP (wide: 16.92 ± 5.46 Nm/kg, TO: 16.49 ± 4.07 Nm/kg, REF: 10.25 ± 4.14 Nm/kg *p* < 0.01, effect size: η2 = 0.406, observed power = 0.983) and vertical directions (wide: 0.32 ± 0.02 Nm/kg, TO: 0.29 ± 0.02 Nm/kg, REF: 0.17 ± 0.02 Nm/kg *p* < 0.01, effect size: η2 = 0.430, observed power = 0.904) (Fig 7, middle panel (B)) (Table 3).

**Table 3:** Joint work and postural sway during stand-to-sit (StandTS).

IFP	Knee Joint Work (Nm/kg) A-P	Knee Joint Work (Nm/kg) Vertical	Hip Joint Work (Nm/kg) A-P	Hip Joint Work (Nm/kg) Vertical	Postural sway (m/s) A-P	Postural sway (m/s) M-L	Postural sway (m/s^2^) Vertical
**REF**	1.08 ± 0.41	0.02 ± 0.01	10.25 ± 4.14*	0.17 ± 0.02*	0.076 ± 0.021	0.092 ± 0.029*	0.97 ± 0.26*
**Wide**	1.50 ± 0.69	0.02 ± 0.01	16.92 ± 5.46	0.32 ± 0.02	0.073 ± 0.028	0.064 ± 0.019	0.68 ± 0.24
**TO**	1.34 ± 0.49	0.03 ± 0.01	16.49 ± 4.07	0.29 ± 0.02	0.056 ± 0.016	0.067 ± 0.020	0.69 ± 0.29

Values are presented as mean ± standard deviation.

* represents a significant difference from the other two IFPs. A-P (anterior-posterior), M-L (medio-lateral).

### Postural sway

In the ML and vertical directions, a significantly reduced postural sway was observed in the wide and TO compared to the REF IFP (The mean velocity of CoP in ML direction: wide = 0.064 ± 0.019 m/s, TO = 0.067 ± 0.020 m/s, REF = 0.092 ± 0.029 m/s, *p* < 0.01, effect size: η2 = 0.345, observed power = 0.94, Vertical direction: wide = 0.68 ± 0.24 m/s, TO = 0.69 ± 0.29 m/s, *p* = 0.01, effect size: η2 = 0.403, observed power = 0.700). There was no difference in postural sway in the AP direction among IFPs (Fig 7, bottom panel (C)) (Table 3).

### Muscle synergy

**Fig 8:**
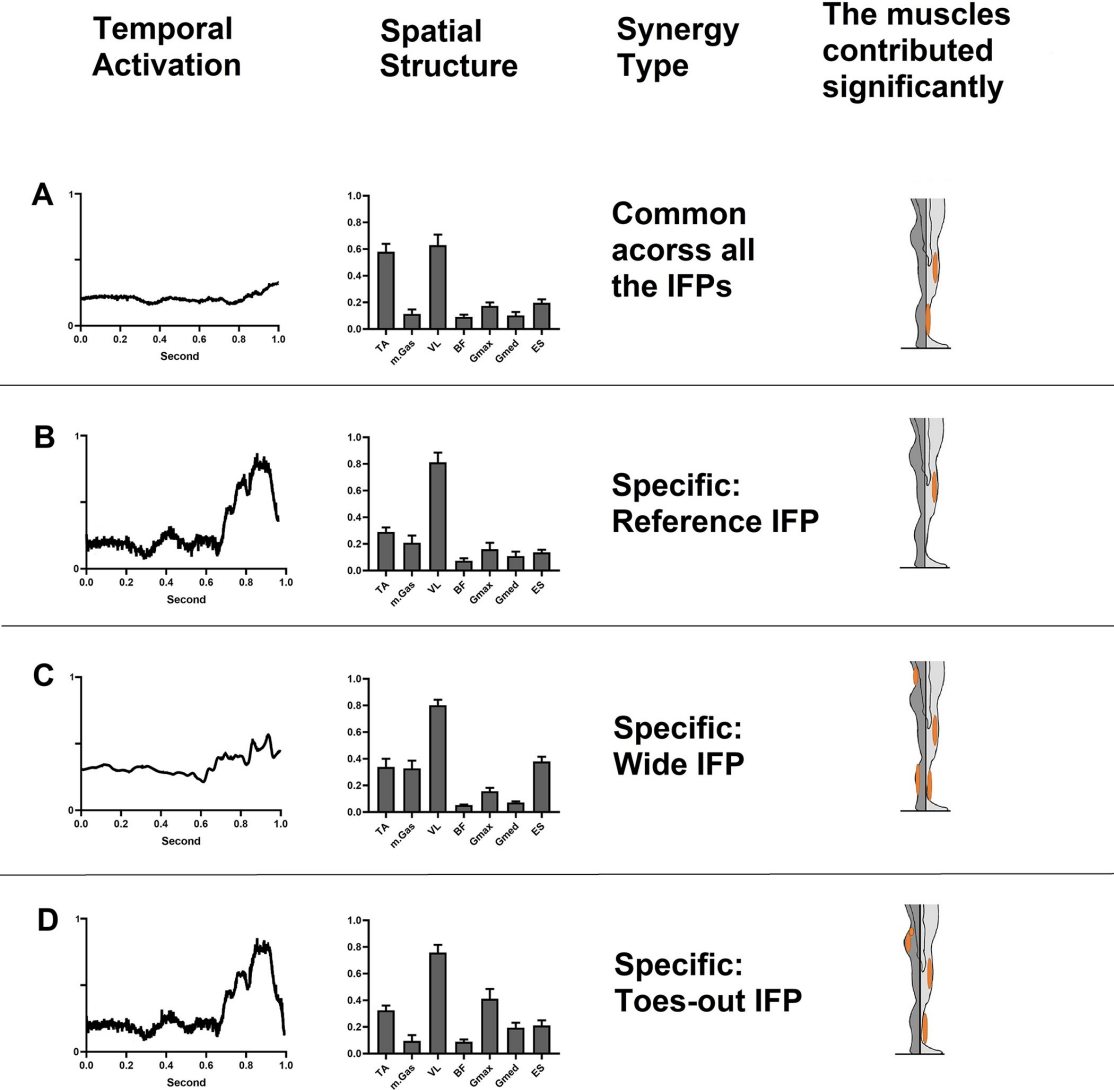
Common and specific muscle synergies of REF, wide, and TO IFPs during the descending phase of StandTS. The left column displays the temporal recruitment coefficient. The middle column showcases the spatial component, indicating the relative contribution of each muscle. The third column indicates synergy type (common or specific) and right column highlights the predominant muscle within the spatial synergy component.

There was no significant difference in the mean value of the number of synergies extracted between the three IFP (REF 2.5 ± 0.67, wide 2.73 ± 0.66, TO 2.87 ± 0.58). Muscle synergies from all participants were grouped into 4 clusters (see Fig 8). Within these clusters, both common synergy across IFPs and IFP-specific muscle synergies were identified.

A cluster showing good reliability (ICC value ≥ 0.75) consistently across all IFPs was observed. This common synergy was characterized by predominant TA and VL muscles activation (Fig 8, top panel (A)). IFP-specific muscle synergies displayed excellent reliability (ICC value ≥ 0.90). The REF specific muscle synergy cluster had prominent VL muscle activation. The wide specific muscle synergy cluster showed a greater contribution of mGas and ES, along with VL (Fig 8, third panel (C)). The TO specific muscle synergy cluster exhibited relatively greater contributions from Gmax and Gmed, along with VL (Fig 8, bottom panel (D)).

## Discussion

This study provides a comprehensive analysis of how different IFPs—REF, wide, and TO—influence joint mechanics, postural control, and muscle activation during the descending phase of StandTS movement. We found significant differences in biomechanical and neuromuscular variations across different IFPs, with implications for optimizing movement control and stability in rehabilitation settings.

### Joint biomechanics and muscle activation patterns

Our results indicate that the TO IFP enhances dorsiflexion and knee flexion compared to REF and wide IFPs. The greater dorsiflexion and knee flexion observed in the TO position are consistent with the idea that an external foot rotation facilitates the ankle and knee joint movements by reducing impingement at the ankle and aligning the knee joint more optimally [33,34]. TO feet positioning likely reduces soft tissue tightness (shortening of the muscles, tendons, or ligaments) around the ankle during dorsiflexion, thereby creating more space for ankle joint movement compared to parallel feet positioning (REF) [35]. Furthermore, this position decreases tension in the lateral knee structures such as the iliotibial band and lateral collateral ligament, promoting more efficient knee flexion [36,37]. These biomechanical adjustments appear to enhance the range of motion for sitting down and contribute to smoother transitions during StandTS by reducing joint strain and facilitating better control over the center of gravity.

Interestingly, while there were no significant differences in hip flexion angles among the IFPs, the wide IFP demonstrated a reduced trunk angle during the descent. Another notable characteristic we observed in wide IFP was an increased knee valgus angle, where the knees move inward toward the midline of the body during the StandTS descent. Wide feet positioning can influence knee and hip alignment, as it leads to hip abduction (outward movement) and external rotation [38], thereby increasing valgus angle at the knee joint.

This altered alignment places the hip adductor muscles in a position where they need to work harder to prevent excessive stress on the knee joint and stabilize the hip joint during StandTS movement. Therefore, increasing tension in the adductor muscles can restrict the range of motion of the trunk joint [39,40]. Consequently, the mechanics of the hip joint are altered by the increased valgus angle in the wide IFP, and this limits the range of motion available for trunk flexion during the StandTS descent. In contrast, a toes-out stance allows the knees to track in the same direction as the toes. This alignment can encourage proper knee movement during the descending phase without placing additional tension on the adductor muscles. Furthermore, a toes-out position helps maintain a neutral pelvic position, promoting optimal hip alignment [41]. This can reduce the likelihood of excessive anterior pelvic tilt, which may strain the lower back and trunk muscles. Since the squat shares a similar descending phase with the StandTS movement, the TO IFP promotes proper knee alignment, providing a more comfortable experience and reducing the risk of inward knee collapse (valgus collapse), which can lead to knee pain or injury.

Additionally, we demonstrated that sitting down with a wider stance requires more activation of the back muscle (erector spinae) and plantar flexor (m. gastrocnemius muscle). From a biomechanical perspective, this observation suggests compensation for the unstable posture in the AP direction during the descending phase of StandTS. In wide IFP, the CoM is not fully moved backward due to limited trunk flexion, resulting in the CoM being positioned relatively forward of the base of support (BoS). To prevent the CoM from crossing over the anterior limit of the BoS, increased action of the plantar flexion is required to shift the CoM backward, counteracting excessive dorsiflexion. Concurrently, the erector spinae muscles maintain an upright trunk position to stabilize the CoM within the anterior boarder of the BoS.

Indeed, IFP-specific muscle synergy analysis reinforces our findings, showing that the wide-specific muscle synergy exhibited a greater contribution from the mGas and ES, along with VL (see Fig 8, third panel (C)). While both mGas and ES muscles contribute to balancing the CoM, their role during StandTS descent is not to actively move the CoM posteriorly. Instead, these muscles function to prevent the CoM moving forward, thereby maintaining the CoM over BoS.

### Eccentric work at the knee and hip joints and muscle activation patterns

Work at the knee and hip joints during StandTS can be divided into two components based on the direction of force during StandTS. Negative eccentric work in the AP direction absorbs ground impact [42,43], while positive eccentric work in vertical direction resists gravity to control the smoothness of descent and minimize abrupt downward movements during StandTS [10]. Eccentric work at the knee and hip joints in both directions is crucial for controlling balance during the sitting down phase of StandTS, ensuring a safe landing.

While there were no differences in knee joint eccentric work across IFPs (REF, wide, and TO), eccentric work at the hip joint demonstrated that the wide and TO IFPs created greater eccentric work in both the AP and vertical directions compared to REF. Similar to knee extensor eccentric control, hip extensor (Gmax) eccentric control decelerates the body’s CoM, ensuring a smooth and controlled transition from standing to sitting. Particularly, eccentric hip extensor activity collaborates with the erector spinae muscles to prevent excessive forward lean and potential balance loss [10]. Additionally, proper eccentric work at the hip joint during the descent motion ensures even force distribution across the hip and knee joints [44], thereby reducing excessive stress at the knee joint and lowering the risk of fall-related injuries during StandTS.

Postural sway results further highlight the active role of increased eccentric work by the hip extensors in both the wide and TO IFPs. Both the wide and TO IFPs significantly reduced postural sway in the vertical and ML directions compared to the REF position. The reduction in vertical postural sway suggests that wide and TO IFPs facilitate easier eccentric contraction of the hip extensors, effectively resisting the pull of gravity. Specifically, the TO-specific muscle synergy analysis showed a greater contribution from the Gmax and Gmed, along with VL (see Fig 8, bottom panel (D)).

Excessive hip flexion during StandTS without sufficient eccentric control by hip joint muscles can lead to loss of balance or uncontrolled descent from standing. Activating the hip extensors stabilizes the pelvis and counteracts the rapid motion of core muscles and trunk flexion, ensuring a smooth descent [45]. Our findings support the advantage of a TO IFP for squat rehabilitation. The TO stance promotes greater activation of the Gmax and Gmed, which are essential for stabilizing the pelvis and hips during squats. This enhanced activation improves strength and provides a greater sense of stability, resulting in a more comfortable balanced squat experience.

Additionally, reduced postural sway in the ML direction for wide and TO IFPs indicates enhanced lateral stability. These findings are consistent with previous research showing that wider stances can enhance lateral balance control by increasing the BoS and reducing the lateral movement of the CoP [4,46].

### Clinical reflection

The findings from this study highlight the importance of initial foot positioning in optimizing biomechanical efficiency and neuromuscular control during the StandTS movement. These insights have practical implications for tailoring rehabilitation strategies to specific patient needs.

We observed that wide IFP limits trunk flexion and engages the back muscles more effectively. This foot positioning is particularly advantageous for patients experiencing back pain, as it reduces the demand on the trunk flexors and enhances the involvement of stabilizing back muscles. The wide IFP can be integrated into squat rehabilitation exercises for patients with back pain. This foot position helps patients perform movements with reduced trunk flexion, thereby minimizing the risk of aggravating back pain during the squat exercise and promoting the engagement of stabilizing muscles in the back. Additionally, patients in the early stages of Parkinson’s disease often exhibit postural changes, including forward trunk lean (stooped posture) and increased lordosis [47], partially due to decreased motor activation of the paraspinal muscles. Prescribing the wide IFP for StandTS may help increase recruitment of the erector spinae, reducing atrophy over time and potentially slowing the disease’s hallmark postural changes [48].

Our findings demonstrated that TO IFP promotes greater joint flexibility at the ankle and knee and enhances the contribution of the hip extensor muscles. This foot position is particularly beneficial for patients with conditions that require reducing load concentration on the knee joint and improving joint flexibility. Incorporating TO IFP into Sit-to-Stand and Stand-to-Sit exercise in rehabilitation program for arthritis patients can help improve their joint flexibility and hip muscle strength while reducing knee strain and discomfort.

## Conclusions

Overall, our results suggest that adjusting initial foot positioning can significantly impact the biomechanics and neuromuscular control during the StandTS movement. Compared to the REF IFP, both wide and TO IFPs enhance hip joint work, improving balance control during the transition to sitting. The wide IFP increases valgus angle at the start of sitting down, leading to reduced trunk flexion and greater engagement of back muscles during the StandTS descent. The TO IFP allows for greater angular displacement at the ankle and knee joints, increasing joint flexibility and enhancing hip joint muscle involvement for better control of StandTS movement control. These findings provide valuable insights for designing rehabilitation strategies tailored to specific patient needs.

## Supporting information

S1 File (DOCX)
