# Supplementary material for: Effects of initial foot position on neuromuscular and biomechanical control during the stand-to-sit movement: Implications for rehabilitation strategies
Source: PLoS One. 2025 Feb 14;20(2):e0315738. doi: 10.1371/journal.pone.0315738 (PMC11828351; doi:10.1371/journal.pone.0315738)
Supplement: S1 File — (DOCX) [file pone.0315738.s001.docx]

JOURNAL REQUIREMENTS:

1. Please ensure that the author list and affiliations are correct on the title page of your manuscript, and that your author contributions, competing interests, and financial disclosure are correct as listed below. All of these sections will be indexed in PubMed and published by PLOS ONE as you have written them. Please email plosone@plos.org if any changes to this content need to be made.

Woohyoung Jeon:
Conceptualization
Data curation
Formal analysis
Investigation
Methodology
Project administration
Resources
Software
Supervision
Validation
Visualization
Writing – original draft
Writing – review & editing

Ashley Dalby:
Conceptualization
Data curation
Investigation
Methodology
Validation
Writing – review & editing

Xuanliang Neil Dong:
Conceptualization
Formal analysis
Investigation
Methodology
Software
Writing – review & editing

Chung-Hyun Goh:
Conceptualization
Investigation
Methodology
Software
Writing – review & editing


Please see here for the full list and definition of contributor roles: http://journals.plos.org/plosone/s/authorship#loc-author-contributions

This is correct.

2. Please ensure that the Competing Interests and Financial Disclosure statements listed below are suitable for publication. These sections will be indexed in PubMed and published by PLOS ONE as you have written them. Please email plosone@plos.org if any changes to these statements need to be made.

Competing Interests:

The authors have declared that no competing interests exist.

Financial Disclosure:

The author(s) received no specific funding for this work.

This is correct.

3. "In your Data Availability statement, you have noted that you will provide repository information for your data at acceptance. Now that your manuscript has been accepted for publication, please upload the minimal anonymized dataset necessary to replicate your study findings to a stable, public repository and provide the accession numbers or DOIs necessary to access these data. You also have the option of uploading the data as Supporting Information files, but we would recommend depositing data directly to a data repository if possible.

Please provide a revised Data Availability Statement and be sure to include:

A. Location of the data (i.e. the repository name) Zenodo.org
B. The DOI/accession number of each dataset AND/OR a direct link to freely access each data set. DOI [10.5281/zenodo.14285541](https://doi.org/10.5281/zenodo.14285541).

If you are unable to adhere to our open data policy, please kindly revise your statement to explain your reasoning and we will seek the editor's input on an exemption. Your paper will be held at this point until a resolution is reached, and this could add significant delay to the publication schedule. Our full policy is located here: https://journals.plos.org/plosone/s/data-availability"

4. To prevent production delays, we recommend using the Author Formatting Checklist to confirm that your paper meets PLOS ONE's typesetting requirements for References, Tables, and Figures: <http://journals.plos.org/plosone/s/file?id=c819/plos-one-author-formatting-checklist.docx>. This has been checked.

This checklist is a reference tool for you; please do not upload the completed Author Formatting Checklist with your submission files.

5.To ensure your figures meet our technical requirements, please run each figure included in your submission files through the PACE tool: https://pacev2.apexcovantage.com/. PACE will assess whether your figures meet our technical requirements and will fix the figure(s) or identify any problem(s) that cannot be automatically fixed. It can also convert figures to TIFF format, resize, and rename figures to meet our naming conventions.
To use PACE, first register as a user. Follow the instructions on the site for assessing and converting your figure files. If you experience any difficulty using this tool or have questions about any of the figures and/or images in your paper, please inform the journal office in your response letter. This has been checked with the original figures.

CONFIDENTIAL: This email and any attachments are confidential and for the sole use of the individual(s) to whom they are addressed. If you have received this message in error please delete the message and notify plosone@plos.org.

__________________________________________________
In compliance with data protection regulations, you may request that we remove your personal registration details at any time. (Use the following URL: https://www.editorialmanager.com/pone/login.asp?a=r). Please contact the publication office if you have any questions.
